# Bone Marrow Mononuclear Cells Combined with Beta-Tricalcium Phosphate Granules for Alveolar Cleft Repair: A 12-Month Clinical Study

**DOI:** 10.1038/s41598-017-12602-1

**Published:** 2017-10-23

**Authors:** Fengzhou Du, Huanhuan Wu, Haidong Li, Lei Cai, Qian Wang, Xia Liu, Ran Xiao, Ningbei Yin, Yilin Cao

**Affiliations:** 1 Research Center of Plastic Surgery Hospital, Chinese Academy of Medical Sciences & Peking Union Medical College, Beijing, P.R. China; 2 Cleft Lip and Palate Center, Plastic Surgery Hospital, Chinese Academy of Medical Sciences & Peking Union Medical College, Beijing, P.R. China

## Abstract

Alveolar cleft is the most common congenital bone defect. Autologous iliac crest bone graft (ICBG) is the most widely adopted procedure for alveolar cleft repair, but the condition is associated with door-site morbidities. For the first time, this study used bone marrow mononuclear cells (BMMNCs) combined with beta-tricalcium phosphate (β-TCP) granules to repair alveolar bone defect. The effectiveness of this technique was compared with autologous ICBG after 12 months of follow-up. The bone formation volume was quantitatively evaluated by three-dimensional computed tomography and computer aided engineering technology. BMMNCs/β-TCP granule grafting was radiographically equivalent to ICBG in alveolar cleft repair. Although considerable resorption was observed up to 6 months after surgery, no significant differences were noted in the Chelsea score and bone formation volume between groups. These finding indicate that BMMNCs/β-TCP grafting is a safe and effective approach for alveolar bone regeneration.

## Introduction

Alveolar cleft is the most common congenital bone defect. Secondary alveolar bone grafting prior to canine eruption is considered the standard procedure for patients with cleft lip and palate^1,2^. Repair of the alveolar bone cleft helps to restore dental arch continuity, stabilize the maxilla, facilitate subsequent orthodontic treatment, and provide support to soft tissue structures^3,4^. Iliac crest bone graft (ICBG) is the “gold standard” for the repair of alveolar cleft^1,5–7^. However, ICBG is associated with complications, such as severe postoperative pain at the donor site, pelvic instability, nerve injury and infection^8–10^. Additionally, the success rate of alveolar cleft repair with ICBG varies considerably in different reports^1,11–13^.

To avoid the above disadvantages, new strategies for bone regeneration have been sought, such as osteo-conductive biomaterials, cytokines, and most notably bone morphogenetic proteins (BMPs)^14–16^. Stem cell therapy is a promising alternative to autologous bone grafting. A combination of cultured mesenchymal stem cells (MSCs) and biomaterials is a common strategy in bone defect repair, and its effectiveness has been demonstrated in various animal models^17–20^. However, preparation of cultured grafts involves a complicated procedure, high manufacturing costs and a risk of contamination, which makes it very difficult to obtain regulatory approval for clinical application. To date, very few clinical studies using *ex vivo* culture-expanded MSCs have been reported^21,22^.

Bone marrow is a rich source of MSCs. Bone marrow mononuclear cells (BMMNCs) contain numerous regenerative cells and can be prepared during the surgical procedure. Previously, BMMNCs have been used in spine fusion and bone non-union but rarely in craniofacial bone defect repairing^23,24^. In this study, BMMNCs were combined with beta-tricalcium phosphate (β-TCP) granules to repair alveolar bone defects in cleft lip patients for the first time. The study compared the effectiveness of BMMNCs/β-TCP with standard ICBG. Bone formation was quantitatively evaluated by three-dimensional (3D) computed tomographic (CT) scans and computer-aided engineering (CAE) during a 12-month follow-up period.

## Results

### Clinical Outcomes

Twenty unilateral cleft lip (UCL) patients were enrolled in the study, all of whom had received primary modified Millard cleft lip repair at an early age. The age range of the patients was 8 to 28 years. Of the 20 patients, 10 chose BMMNCs/β-TCP graft, whereas the remaining 10 patients underwent iliac crest bone grafting. The main outcomes of the study are summarized in Tables 1 and 2. In the BMMNCs/β-TCP group, three patients were unable to schedule a follow-up at 3 and 12 months postoperatively, and one patient missed the 6-month follow-up. In the ICBG group, one patient missed follow-up at 6 months postoperatively, and another patient missed the 12-month follow-up.

**Table 1:** Group1: bone marrow mononuclear cells combined with beta-tricalcium phosphate granules.

Patient	Age (yr)	Sex	Alveolar Cleft	Defect Volume (mm^3^)	3 Months Postoperation		6 Months Postoperation		12 Months Postoperation		Bone union	Chelsea Scale	Duration of Hospital Stay (days)
					Bone Volume (mm^3^)	Bone Formation Ratio(%)	Bone Volume (mm^3^)	Bone Formation Ratio(%)	Bone Volume (mm^3^)	Bone Formation Ratio(%)			
1	12	M	Unilateral, R	1188.9	969.6	81.6	786.5	66.2	743.6	62.5	Yes	8	3
2	11	F	Unilateral, L	631.5	491.0	77.8	310.3	49.1	—	—	Yes	4	3
3	11	F	Unilateral, L	925.5	655.8	70.9	473.1	51.1	438.1	47.3	Yes	8	3
4	10	F	Unilateral, L	1174.7	—	—	854.8	72.8	827.3	70.4	Yes	8	3
5	16	M	Unilateral, L	885.8	—	—	—	—	384.3	43.4	No	4	3
6	10	M	Unilateral, R	1351.6	1073.6	79.4	722.8	53.5	707.4	52.3	Yes	8	4
7	8	M	Unilateral, R	764.1	568.6	74.4	433.2	56.7	401.7	52.6	Yes	6	3
8	8	F	Unilateral, R	967.8	564.3	58.3	400.2	41.4	—	—	Yes	5	3
9	28	F	Unilateral, R	1155.1	—	—	870.5	75.4	—	—	No	4	3
10	11	M	Unilateral, R	1116.1	748.4	67.1	658.4	59.0	620.7	55.6	Yes	7	4

M, male; F, female; R, right side; L, left side.

**Table 2:** Group2: iliac crest bone graft.

Patient	Age (yr)	Sex	Alveolar Cleft	Defect Volume (mm^3^)	6 Months Postoperation		12 Months Postoperation		Bone Union	Chelsea Scale	Duration of Hospital Stay (days)
					Bone Volume (mm^3^	Bone Formation Ratio(%)	Bone Volume (mm^3^)	Bone Formation Ratio(%)			
1	15	F	Unilateral, R	1090.5	716.4	65.7	700.5	64.2	Yes	8	6
2	11	F	Unilateral, L	1131.2	170.3	15.1	160.5	14.2	No	3	6
3	9	F	Unilateral, L	1125.9	740.1	65.7	736.0	65.4	Yes	8	6
4	8	M	Unilateral, L	820.9	775.3	94.4	753.5	91.8	Yes	7.5	5
5	12	F	Unilateral, R	1319.6	854.6	64.8	815.2	61.8	Yes	8	6
6	14	M	Unilateral, R	1073.2	—	—	616.2	57.4	Yes	5	6
7	9	F	Unilateral, R	1187.1	794.1	66.9	748.2	63.0	Yes	8	5
8	11	F	Unilateral, L	1146.2	694.5	60.6	662.3	57.8	Yes	7	6
9	11	F	Unilateral, R	1296.0	820.3	63.3	—	—	Yes	8	6
10	11	M	Unilateral, R	1078.5	542.5	50.3	525.4	48.7	No	4	5

M, male; F, female; R, right side; L, left side.

All patients healed properly without any infection. Patients from the BMMNCs/β-TCP group could ambulate immediately after surgery and did not report any donor site discomfort during the 12-month follow-up. In the ICBG group, patients were required to have bed rest for the first 3 days postoperatively. All of the patients complained of moderate donor site pain when discharged, and approximately one-third of patients (3/10) reported tenderness of the iliac crest up to 6 months after surgery. Moreover, the duration of hospital stay in the ICBG group was considerably increased compared with the BMMNCs/β-TCP group.

### Radiographic Evaluation

**Figure 1:**
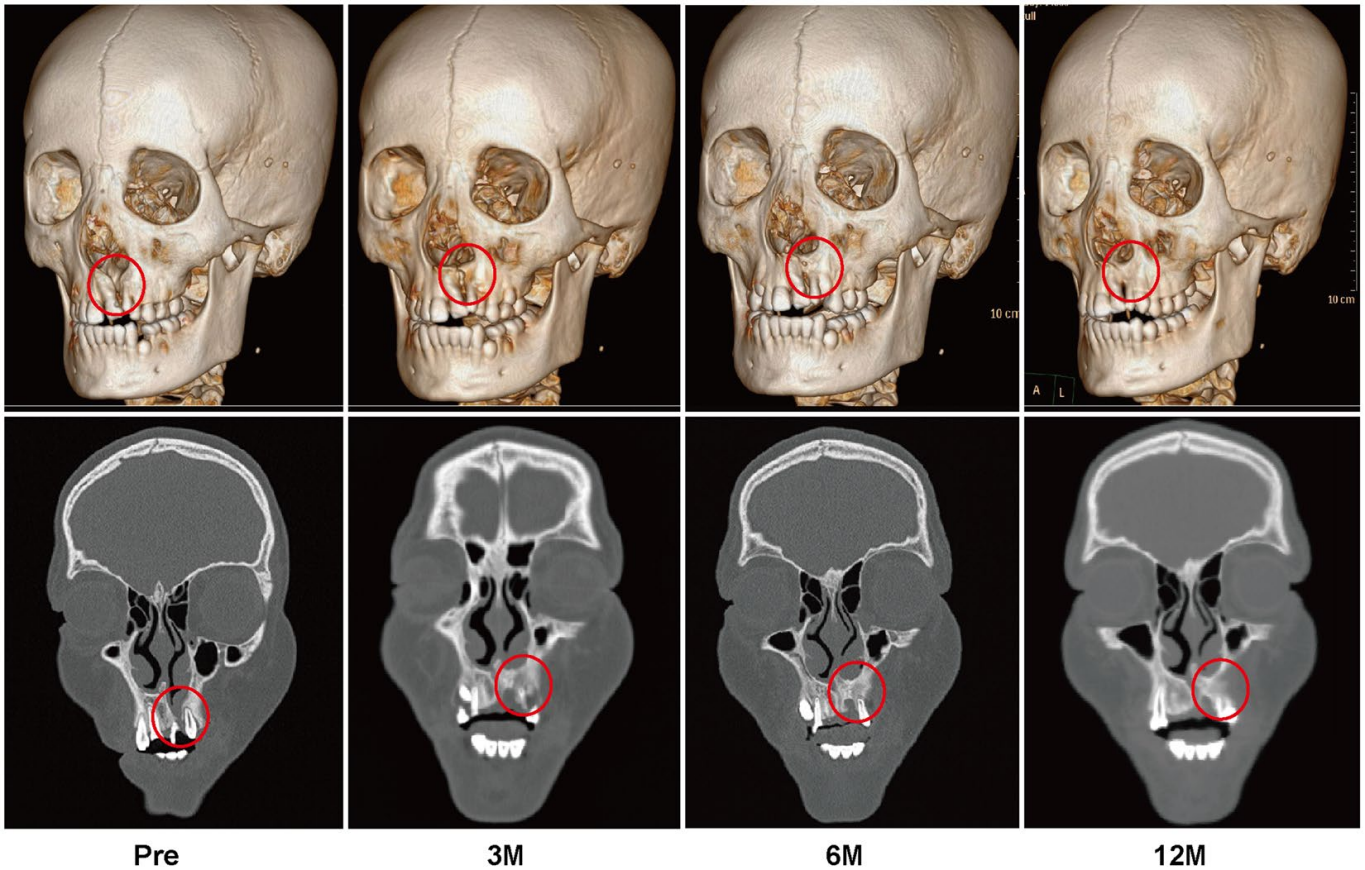
Typical three-dimensional and coronal computed tomographic images of a patient who underwent BMMNCs/β-TCP grafting. From left to right, images taken preoperatively and at 3, 6, and 12 months postoperatively.

**Figure 2:**
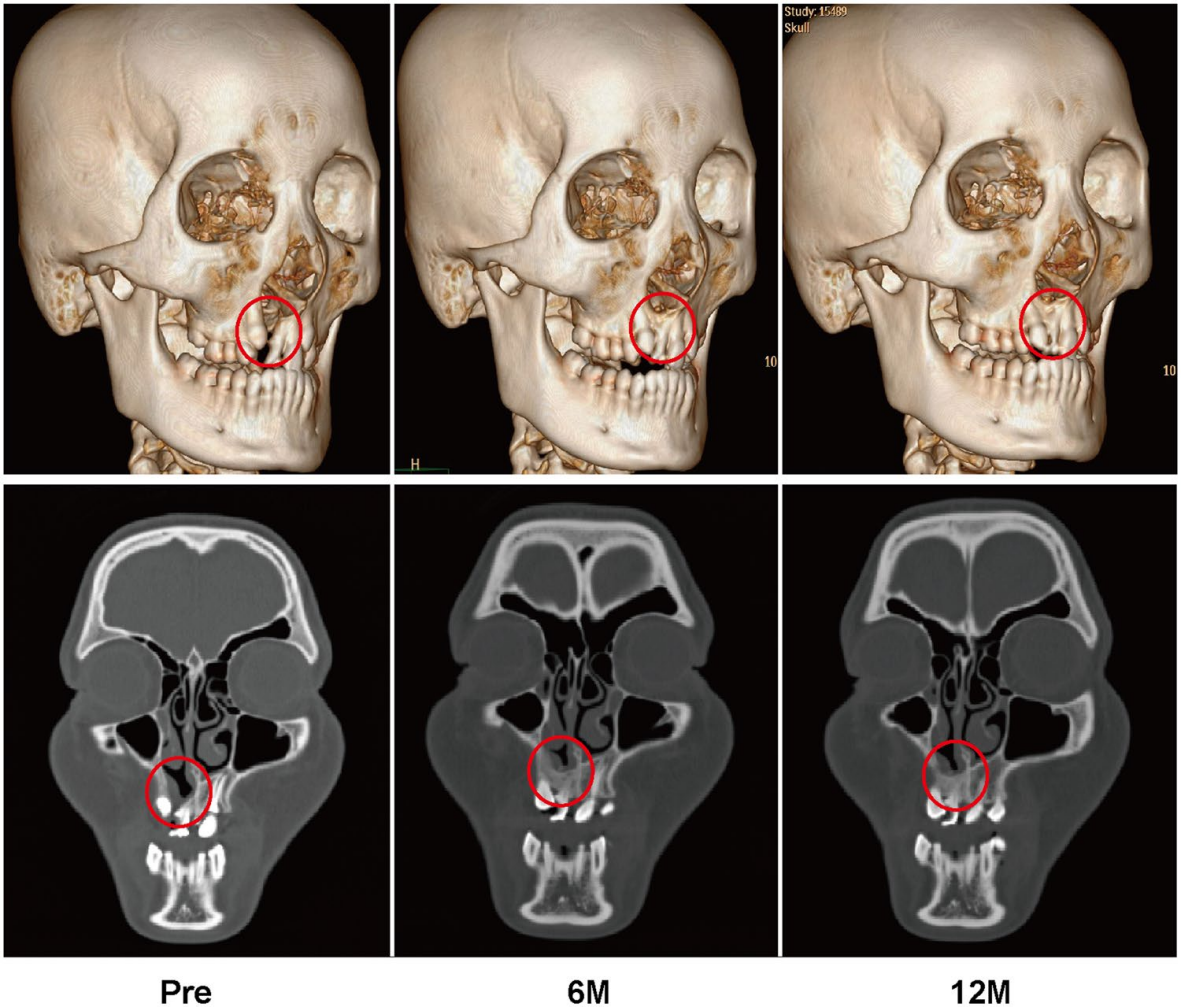
Typical three-dimensional and coronal computed tomographic images of a patient who underwent ICBG grafting. From left to right, images taken preoperatively and at 6 and 12 months postoperatively.

Alveolar bone union was achieved in the majority of patients in the BMMNCs/β-TCP and ICBG groups. The three-dimensional and coronal CT scans of a 12-year-old boy from the BMMNCs/β-TCP group and a 13-year-old girl from the ICBG group at different follow-up time points are presented in Figs 1 and 2, respectively. The alveolar bone defects were repaired in both cases. However, in certain cases, the alveolar defects were not completely repaired in both BMMNCs/β-TCP and ICBG groups (Tables 1 and 2). In the BMMNCs/β-TCP group, two patients’ clefts were only partly repaired, and the dental arches were not restored. In the ICBG group, one patient exhibited partial repair, and another patient’s graft was almost completely absorbed 6 months after surgery.

**Figure 3:**
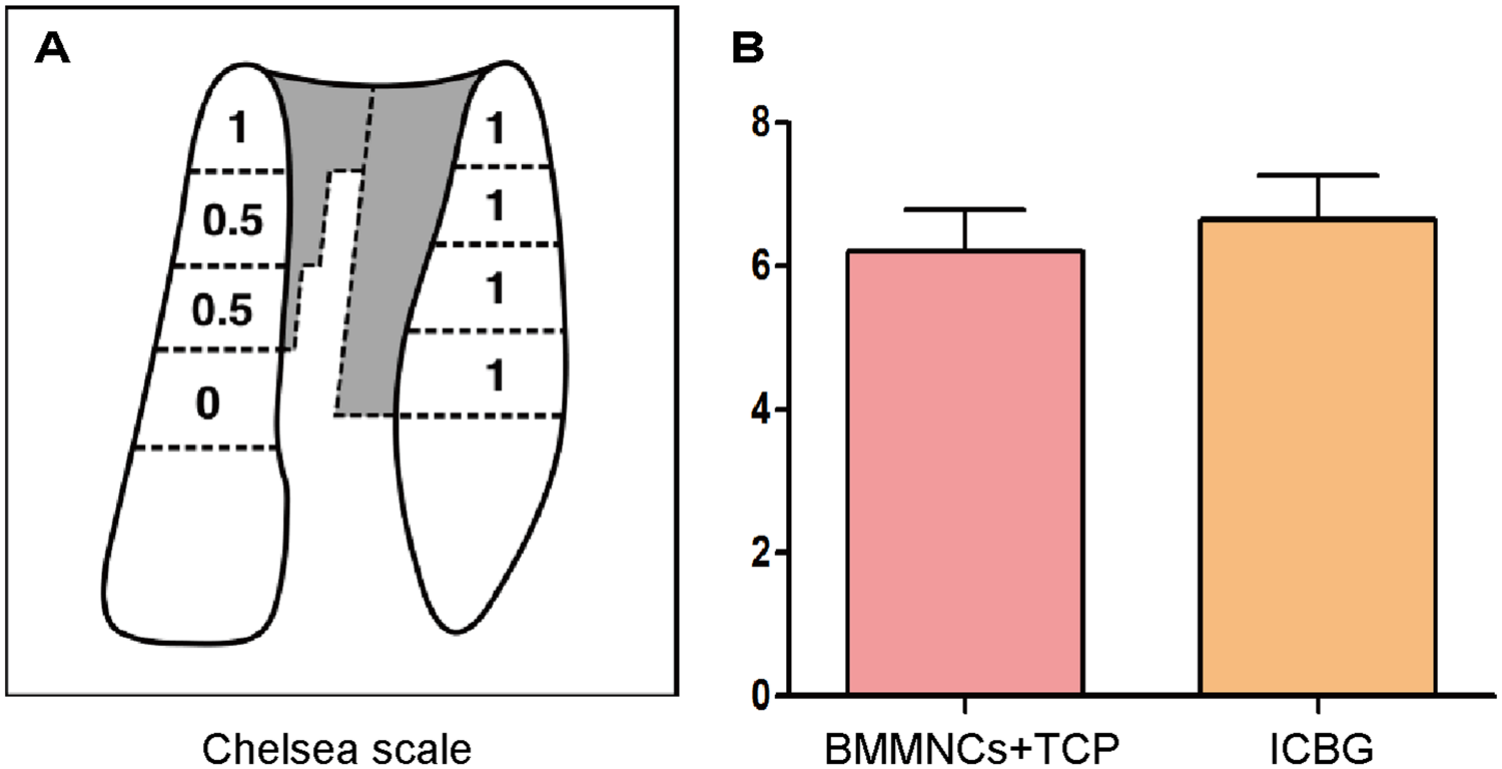
Chelsea scale analysis. (**A**) Chelsea scale quantifies bone formation at the cleft site using a score that ranges from 0 to 8. Note, a score of 0.5 indicate a partial fill without bony bridge. (**B**) Comparison of mean Chelsea scores based on procedure. The mean scores of the BMMNCs/β-TCP and ICBG groups are 6.2 ± 0.57 and 6.7 ± 0.61, respectively. The difference is not statistically significant.

The radiographic score was evaluated at the last follow-up point using the 8-point Chelsea Scale (Fig. 3). The mean Chelsea score of the BMMNCs/β-TCP group was 6.2 ± 0.57, which was similar to the ICBG group (6.7 ± 0.61). No significant difference was found between the two groups.

### Quantitative Bone Formation Analysis

**Figure 4:**
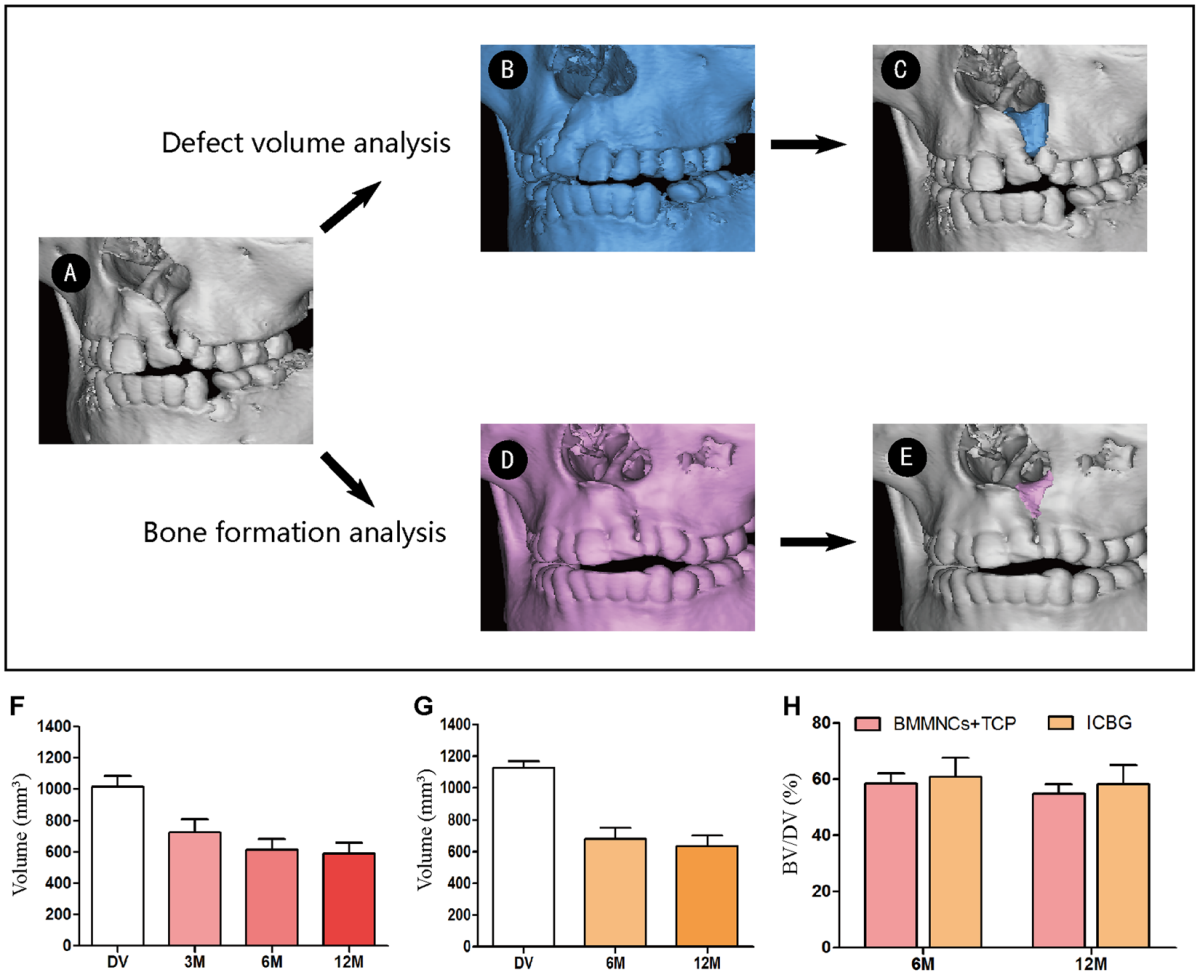
Volumetric analysis using CAE. (**A**) Non-cleft side data were mirrored (blue), and then the original cleft data were subtracted to identify cleft morphology and volume. Postoperative data (pink) was registered with the original cleft to identify the graft and newly formed alveolar bone. Postoperative graft volume changes of patients who underwent BMMNCs/β-TCP and ICBG grafting are presented in (**B**) and (**C**), respectively. (**D**) Bone formation ratio of the two groups at 6 and 12 months postoperatively. DV, defect volume; BV, bone volume.

The CT scan data of both groups were processed with CAE software Mimics 16.0 (Materialise, Leuven, Belgium) and Geomagic Studio 2013 (Geomagic, Morrisville, USA) (Fig. 4). The average defect volume was 1016.1 ± 220.1 mm^3^ and 1126.9 ± 137.4 mm^3^ for the BMMNCs/β-TCP group and ICBG group, respectively. The difference was not significant. New bone volume at the cleft site was analyzed at each follow-up time point. Three months after surgery, the mean bone volume (BV) of the BMMNCs/β-TCP group was 724.5 ± 220.5 mm^3^, and the mean bone formation ratio (BF%) with regard to defect volume was 72.8 ± 3.1%. The mean BV of the BMMNCs/β-TCP group decreased at 6 months after surgery (612.2 ± 211.5 mm^3^), resulting in a significantly reduced mean BF% of 58.4 ± 3.8% (*p* = 0.01). The mean BV at 12-month follow-up had no significant change (589.0 ± 180.5 mm^3^) with a mean BF% of 54.9 ± 3.6%. In the ICBG group, the mean BV was 678.7 ± 211.0 mm^3^ and the mean BF% was 60.8 ± 6.9% 6 months after surgery. Similar to the BMMNCs/β-TCP group, the mean BV of the ICBG group slightly decreased 12 months after surgery (635.3 ± 197.5 mm^3^), corresponding to a mean BF% of 58.3 ± 6.7%. The difference between the two groups was insignificant.

## Discussion

Bone marrow is a natural source of cells and cytokines for bone regeneration. Since 1980s, fresh bone marrow aspirate has been routinely used to repair bone defects. In 1989, Connolly *et al*. reported the centrifugation protocol for BMMNCs preparation^25^. Gupta *et al*. demonstrated that TCP combined BMMNCs had a similar fusion rate and denser bone formation compared with autograft in an ovine lumbar spine fusion model^26^. Muschler *et al*. reported that demineralized bone matrix (DBM) combined with BMMNCs delivered a mean 5.6-fold increase in progenitor cells compared with DBM mixed with bone marrow, resulting in increased spinal fusion volume and union scores^27^.

To our knowledge, this is the first clinical study focusing on alveolar cleft treated with the BMMNC method. Repair of the alveolar cleft involves more than the treatment of a local bony defect. Repair is essential in the sequential treatment of cleft lip and palate^28^. Although iliac crest bone grafting is the most widely adopted procedure, it has a major disadvantage of its associated door-site morbidities. Compared with iliac bone harvest, iliac bone marrow aspiration is a minimally invasive procedure. In this study, we evaluated the efficacy of the BMMNCs/β-TCP granule protocol compared with ICBG, the current standard for alveolar cleft repair. We observed satisfactory results in the majority of patients using BMMNCs/β-TCP scaffold without any complications. No significant differences in the radiographic scores using the Chelsea scale were observed between the BMMNCs/β-TCP and ICBG groups.

BMMNCs contains multiple cell types, among which MSCs are the key component for osteogenesis. MSCs promote bone regeneration by direct osteogenic differentiation, releasing growth factors and stimulation of migration of host osteoprogenitors^29^. With the recent advances in stem cell research, the application of MSCs has advanced from bench to bedside. Zhang *et al*. reported successful repair of created artificial alveolar clefts by bone marrow-derived MSCs in combination with β-TCP scaffolds in a canine model^30^. In 2006, Hibi *et al*. first reported the use of MSCs for clinical alveolar cleft repair in a 9-year-old girl^31^. The BF% of this case is as high as 79.1% after 9 months. Behnia *et al*. used MSCs seeded on DBM and calcium sulfate in 2 patients with alveolar cleft^32^. The 4-month postoperative CT evaluation revealed 34.5% bone formation ratio in one case and 25.6% in the other. In 2012, Behnia *et al*. reported an additional 3 cases^33^. The clefts were repaired by MSCs mounted on biphasic hydroxyapatite/tricalcium phosphate (HA/TCP) scaffold and combined with platelet derived growth factor (PDGF). The BF% was 51.3% after 3 months. Pradel *et al*. used bone marrow-derived osteoblast-like cells cultured on DBM in 4 alveolar cleft patients. The mean BF% was 40.9% after 6 months^34^.

Compared with these case reports, which reported a BF% ranging from 25.6% to 79.1% after 3 to 9 months, the present study quantified bone formation at three time points. The mean BF% of BMMNCs/β-TCP group was 72.8 ± 3.1% 3 months postoperatively, 58.4 ± 3.8% after 6 months and 54.9 ± 3.6% after 12 months. Of note, obvious resorption occurs after bone grafting. Bone graft healing was considered complete 6 months after surgery, which was consistent with previous reports^35^.

BMMNCs contains fewer MSCs than *in vitro* cultured strategies, but much more bioactive molecules and other cells, such as endothelial progenitor cells (EPCs), hematopoietic stem cells (HSCs), platelets, and lymphocytes, are reserved. Studies indicate that a mixture of bone marrow-derived cells may have stronger bone regeneration potential than a single cell type^36^. More importantly, given that BMMNCs can be rapidly produced during operation without further external stimuli, clinical studies were considerably easier to perform.

Very few studies have performed quantitative analysis of bone graft in the alveolar cleft. Although three-dimensional CT enabled a more detailed evaluation of bone formation, analyses are operator dependent, and bone volume information could not be obtained. In this study, both cleft site and bone grafts were segmented accurately using computer-aided engineering, which made it possible to precisely estimate the amount of grafts needed preoperatively and to observe the state of grafts after surgery. Of note, the defect volume calculated by the software provided an ideal value, and repairing an alveolar cleft does not mean complete restoration of the alveolar bone. Most cases satisfied the load-bearing requirements of the tooth and started the orthodontic treatment. The two failed cases were subject to BMMNCs/β-TCP grafting at the ages of 16 and 28, separately. Both of cases exceeded the optimal age for alveolar cleft repair^37,38^. The effectiveness of the BMMNCs/β-TCP approach in adult patients needs further research.

In conclusion, autologous BMMNCs combined with β-TCP granules were radiographically equivalent to ICBG in alveolar cleft repair. Hospital stay days and postoperative pain were reduced significantly. Similar to ICBG, considerable resorption was noted for BMMNCs/β-TCP grafting until 6 months after surgery. Nevertheless, the study demonstrated that BMMNCs/β-TCP grafting was a safe and effective approach for alveolar bone regeneration. The results of the study are promising and encourage larger clinical trials with an expanded patient population.

## Methods

This study was a controlled clinical trial designed to investigate the efficacy of bone marrow mononuclear cells combined with β-TCP granules in alveolar bone defect repair.

### Ethics

The study was approved by the ethics committee of Plastic Surgery Hospital Affiliated to Peking Union Medical College (20121203). All methods were performed in accordance with national and institutional guidelines and regulations of clinical study. The study was registered at Chinese Clinical Trial Registry (ChiCTR) on 21^th^ July 2015 (ChiCTR-ICR-15006799). Written informed consent forms were obtained for all patients or their parents for participation in this study. Another informed consent form for publication of personal information and images was also obtained.

### Patients and Materials

Twenty unilateral cleft lip (UCL) patients were enrolled in the study. All patients enrolled in the study were born with UCL with alveolar bone defect. Exclusion criteria included any previous alveolar surgery, active infection, and underlying diseases, such as hematologic disorders, diabetes, neoplasm and immune deficiency. The patients and their parents were well informed about treatment methods. The patients and their parents were offered two options for alveolar cleft reconstruction: standard alveolar bone grafting with iliac crest bone and the new alternative method using BMMNCs combined with β-TCP.

The micro-structured β-TCP granules (Bio-lu Bioceramics, Bio-lu Biomaterials Co. Ltd, Shanghai, China) have a porosity of 75 ± 10%, and the granule diameter is less than 1 mm.

### Operative Technique

All operations were performed by the same surgical team at Plastic Surgery Hospital, Peking Union Medical College. Under general anesthesia, the gingiva surrounding the alveolar cleft and the soft tissue in superior iliac crest were infiltrated with 0.5% lidocaine with 1:100,000 epinephrine. The intraoral operation and graft preparation were simultaneously performed by two groups of surgeons.

**Figure 5:**
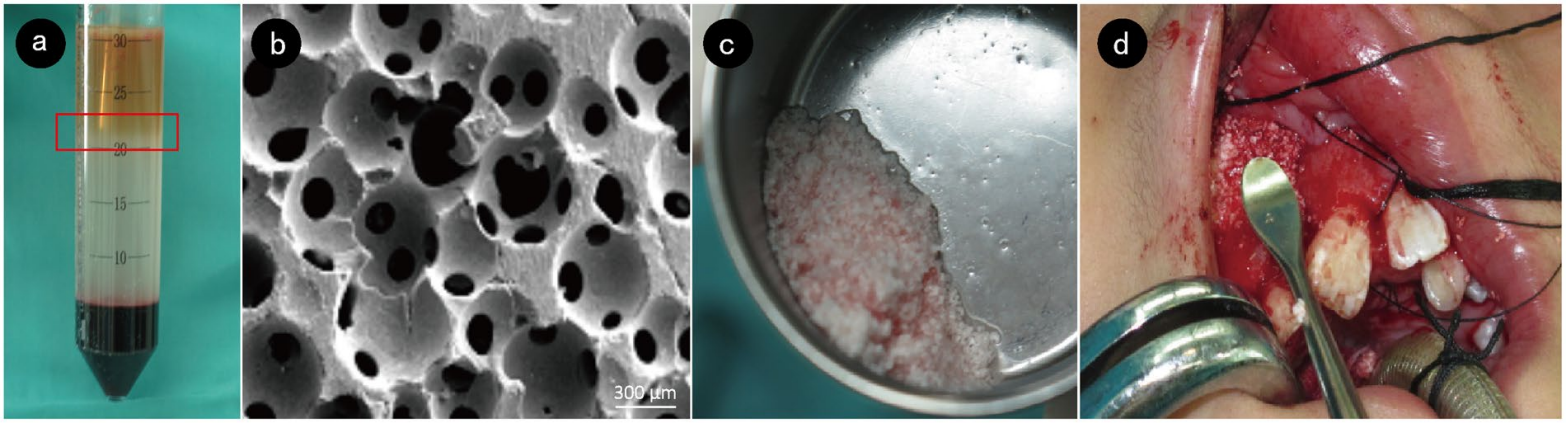
Operative process. (**a**) Bone marrow aspirate after centrifugation. The red frame indicates the layer of BMMNCs. (**b**) Porous structures of β-TCP observed by scanning electron microscope. (**c**) BMMNCs mixed with β-TCP granules. (**d**) Alveolar cleft filled with BMMNCs/β-TCP graft.

Twenty ml of autologous bone marrow was aspirated from the left anterior superior iliac crest through a Klima bone marrow biopsy needle flushed with heparin. To avoid collecting surrounding peripheral blood, the aspiration was performed at 4 different spots around the anterior superior iliac crest as described by Lee and Centeno^39,40^. The aspirated bone marrow was gently transferred into a sterile centrifuge tube filled with 10 ml TBD® Separation Medium (CFDA approved# 2011-1400014, Hao Yang Biological Manufacture Co., LTD, Tianjin, China). Centrifugation was performed in the operating room with no time delay and continued for 15 minutes at 400 g. After centrifugation, approximately 1 ml BMMNCs was aspirated from the layer between the upper plasma and the separation medium. Then, the concentrate was mixed with β-TCP granules. At the alveolar cleft site, gingival sulcus incisions were made on both sides of the cleft. The tissue was then elevated beneath the periosteum. The mucosa of the nasal floor and the oral mucosa were dissected. Next, the bone substitute granules combined with BMMNCs were implanted into the bone defect (Fig. 5). The cleft site was closed without tension by advancement of the gingival flaps.

For the autologous bone graft group, the intraoral surgical procedure was the same as that in the BMMNCs/β-TCP group. At the donor site, a 3-cm incision was made inferior to the superior iliac crest. The subcutaneous tissue was dissected carefully, and the iliac crest was exposed. Cancellous bone was harvested with an osteotome. The cancellous bone particles were cut into small bone particles. The bone graft was transferred to the defect, and the cleft was closed as described previously.

### Postoperative Instructions

All patients were required to eat soft foods and rinse their month after every meal with chlorhexidine mouthwash for 4 weeks. Patients who underwent autologous bone graft were asked to limit their activity for the first three days after surgery.

### Radiographic Assessment

Helical CT scans (Philips, Brilliance CT 128-slice) were obtained preoperatively and at 6 months and 12 months postoperatively from all patients. For patients who underwent BMMNCs/β-TCP treatment, CT scans were also performed 3 months after surgery. Two investigators independently evaluated bone formation according to Chelsea scale at the last follow-up point^41^. The Chelsea Scale is a widely used scoring system for alveolar cleft outcomes based on bone formation position.

CAE technology was adopted for further quantitative evaluation. The DICOM data were processed by Mimics 10.01 (Materialise, Leuven, Belgium). The data were reconstructed as 3D images and saved as standard triangulated language (STL) for further analysis. The newly formed bone was segmented by identifying the differences between preoperative and the postoperative images. Registration was performed on the STL data before and after grafting by Geomagic Studio (Geomagic, Morrisville, USA). Subsequently, the 3D images were superimposed. The newly formed bone in the alveolar cleft could be segmented and the bone volume (BV) was calculated by the software. The morphology of preoperative alveolar clefts varies in different patients. To calculate the bone formation ratio (BF%), the defect volume of each patient was obtained using the method previously described^42,43^. The STL data of the non-cleft side were mirrored to create an ideal maxilla, and the images of the maxilla were then superimposed with the preoperative images to segment the ideal defect volume (DV). BF% was calculated as follows: (BV/DV) × 100% = BF%.

### Statistical analysis

Statistical analysis was performed using the Statistical Package for the Social Sciences 16.0 (Chicago, IL, USA). All values are presented as the mean ± SD. Student’s *t* test was used to analyze the difference between groups and different follow-up points. Values of *p* < 0.05 were considered statistically significant.

## Electronic supplementary material

Study protocol file
