# Supplementary material for: Bone Marrow Mononuclear Cells Combined with Beta-Tricalcium Phosphate Granules for Alveolar Cleft Repair: A 12-Month Clinical Study
Source: Sci Rep. 2017 Oct 23;7:13773. doi: 10.1038/s41598-017-12602-1 (PMC5653813; doi:10.1038/s41598-017-12602-1)
Supplement: Supplementary file 1 — Study protocol file [file 41598_2017_12602_MOESM1_ESM.pdf]

Study Protocol

Objectives

Alveolar cleft is the most common congenital bone defect. Autologous iliac crest bone graft (ICBG) is the most widely adopted procedure for alveolar cleft repairing, but it associates with door-site morbidities. Bone marrow mononuclear cells (BMMNCs) is rich in hematopoietic stem cells, mesenchymal stem cells, endothelial progenitor cells, platelets and a variety of cytokines and other bioactive components, and has been used to assist bone repairing. Beta-tricalcium phosphate is a commercial medical bone repair material that can be completely degraded in the body, and has a good bone conduction effect. In this study, BMMNCs were combined with β-TCP granules to repair alveolar bone defect in cleft lip patients. We intended to explore an effective and propagable technique for the treatment of alveolar defect.

Inclusion criteria

1) 10-40 year old male or female patients with alveolar bone defect;

2) Patients and their families voluntarily underwent surgery, radiological examinations, and follow-up;

3) Patients and their families could read, understand and sign the relevant voluntary informed consent.

Exclusion criteria：

1) Patients participating in other clinical study currently;

2) Patients with obvious abnormality in routine examination;

3) The heart, lungs, brain, kidneys and other vital organ function or hematopoietic function is severely impaired;

4) Patients with obvious malnutrition;

5) Patients with mental and neurological disorders that can not follow instructions of doctors;

6) Recipient site have extensive scarring or poor blood supply caused by extensive radiotherapy or surgery or multiple trauma;

7) Patients received BMP growth factors or other bone growth promoting factor therapy;

8) Patients with a history of metabolic disease that may affect bone regeneration (such as osteoporosis, osteomalacia);

9) Patients received radiotherapy, chemotherapy, immunosuppressive or anticoagulant therapy recently;

10) Patients with systemic or local infection;

11) Patients with allergic constitution or allergic to drugs, regents or materials (TCP) used in the trial;

12) Patients with alcohol or addictive drugs dependence.

Interventions

Group: control group
Sample size: 10
Intervention：Iliac crest bone graft (ICBG)

Group: experiment group
Sample size: 10
Intervention：BMMNCs/β-TCP graft

Study design

Controlled clinical trial

Institution hospital

Plastic Surgery Hospital, Chinsese Academy of Medical Sciences & Peking Union Medical College

Process

Preoperative preparation

☐ Preoperative routine examination

☐ Photograph

☐ Teeth cleaning

☐ Preoperative CT scan

☐ Sign informed consent

☐ Discuss the surgical plan with the patients or their parents

☐ Calculate the volume of alveolar bone defect

Operation

BMMNCs /β-TCP group

Under general anesthesia, the gingiva surrounding the alveolar cleft and the soft tissue in superior iliac crest were infiltrated with 0.5% lidocaine with 1:100,000 epinephrine. The intraoral operation and graft preparation were performed at the same time by two groups of surgeons.

20 ml of autologous bone marrow was aspirated from the left anterior superior iliac crest through a Klima bone marrow biopsy needle flushed with heparin. To avoid collecting surrounding peripheral blood, the aspiration was performed at 4 different spots around the anterior superior iliac crest as described by Lee and Centeno. The aspirated bone marrow was gently transferred into a sterile centrifuge tube filled with 10 ml TBD® Separation Medium (CFDA approved# 2011-1400014, Hao Yang

Biological Manufacture Co., LTD, Tianjin, China). The centrifugation was performed in the operating room with no time delay and continued for 15 minutes at 400g. After centrifugation, about 1 ml BMMNCs were aspirated from the layer between the upper plasma and the separation medium. Then the concentrate was mixed with β-TCP granules. At the alveolar cleft site, gingival sulcus incisions were made on both sides of the cleft. The tissue was then elevated beneath the periosteum. The mucosa of the nasal floor and the oral mucosa were dissected. Next, the bone substitute granules combined with BMMNCs were implanted into the bone defect. The cleft site was closed without tension by advancement of the gingival flaps.

ICBG group

For the autologous bone graft group, the intraoral surgical procedure was the same as the BMMNCs /β-TCP group. At the donor site, a 3 cm incision was made inferior to the superior iliac crest. The subcutaneous tissue was dissected carefully and the iliac crest was exposed. Cancellous bone was harvested with an osteotome. The cancellous bone particles were cut into small bone particles. The bone graft was transferred to the defect, and the cleft was closed.

Postoperative instructions

☐ Soft foods

☐ Rinsing mouth after the meal

☐ For ICBG group, limit the activity for the first three days after surgery

Follow-up

BMMNCs /β-TCP group

CT scan: 3 months, 6 months and 12months after surgery

ICBG group

CT scan: 6 months and 12months after surgery

Outcomes

☐ Bone formation volume

☐ Continuity of alveolar bone

☐ Donor site morbidity
